# Volumetric analysis of hippocampal subregions in migraine without aura: an exploratory study on mechanisms underlying migraine chronification

**DOI:** 10.1186/s10194-025-02183-0

**Published:** 2025-10-31

**Authors:** Qifang Feng, Wen Chen, Jun Ke, Di Geng, Xing Xiong, Lingling Dai, Hongru Zhao, Chunhong Hu

**Affiliations:** 1 Department of Radiology, The First Affiliated Hospital of Soochow University, Shizi Street 188, Suzhou, Jiangsu 215006 People’s Republic of China; 2 Institute of Medical imaging, Soochow University, Soochow, Jiangsu Province People’s Republic of China; 3 Department of Neurology, The First Affiliated Hospital of Soochow University, Shizi Street 188, Suzhou, Jiangsu 215006 People’s Republic of China

**Keywords:** Chronic migraine, Structural neuroimaging, Magnetic resonance imaging, Hippocampus, Subregion

## Abstract

**Background:**

Previous neuroimaging studies on migraine have reported inconsistent hippocampal volumetric changes, potentially due to insufficient consideration of subregion heterogeneity. Exploring the alterations in hippocampal subregions may help reveal the mechanisms underlying migraine chronification. This study aims to investigate the role of volume changes in these subregions in migraine chronification.

**Methods:**

Structural T1-weighted MRI scans were performed on 42 patients with episodic migraine (EM), 22 patients with chronic migraine (CM), and 65 healthy controls (HCs). Hippocampal subregion volumes were quantified using FreeSurfer-based segmentation, and group comparisons were performed with relevant covariates included and false discovery rate (FDR) correction applied to control for multiple comparisons.

**Results:**

No hippocampal subregion volume differences survived FDR correction at 0.05. However, trend-level volumetric variations were observed across the three groups, including the bilateral whole hippocampus, subiculum, cornu ammonis (CA) 1, molecular layer, as well as the left granule cell and molecular layer of the dentate gyrus (GC-ML-DG) and CA4. Subsequent exploratory pairwise comparisons showed that (1) compared with HCs, CM patients exhibited trend-level smaller volumes in the bilateral whole hippocampus, subiculum, CA1, and molecular layer; and (2) compared with EM patients, CM patients showed trend-level reductions in the bilateral whole hippocampus, molecular layer, CA1, as well as in the left subiculum, GC-ML-DG, and CA4.

**Conclusion:**

No significant volumetric differences in hippocampal subregions were detected among the three groups. Exploratory findings suggest trend-level alterations in several hippocampal subregions, including CA1, CA4, molecular layer, GC-ML-DG, and subiculum in CM patients. These preliminary findings may suggest potential links to pain perception, emotion, memory, and cognition during migraine chronification.

**Supplementary Information:**

The online version contains supplementary material available at 10.1186/s10194-025-02183-0.

## Introduction

Migraine is a prevalent and debilitating neurological disorder primarily characterized by unilateral, moderate to severe, pulsating headaches, often accompanied with non-pain symptoms such as nausea, vomiting, photophobia, and phonophobia [1]. It is recognized as the second leading cause of disability worldwide [2], severely impairing the daily life of the patients. Based on the frequency of attacks, migraines are typically classified into episodic migraine (EM) and chronic migraine (CM). It has been shown that 3.4% of EM patients transition to CM within three months [3], and approximately 26% of patients with CM revert to EM over a span of two years [4]. Individuals with CM experience more severe functional impairments, a lower quality of life, and also a greater economic burden [5, 6]. Currently, the pathophysiological mechanisms of migraine chronification have been attributed to inflammation and central sensitization theories [7, 8]; however, the exact mechanisms remain incompletely understood. Further exploration of the neurobiological mechanisms underlying migraine chronification are essential for guiding early clinical diagnosis and precision interventions.

In recent years, significant progress has been made in understanding the mechanisms of migraine chronification through the use of modern neuroimaging techniques. Among these advancements, one brain structure that has gained increasing attention as an important contributor to its pathophysiology is the hippocampus [9–11]. As a key component of the limbic system, the hippocampus is involved in emotional regulation and memory formation, and it also contributes to pain processing and modulation [12, 13]. The volume of hippocampus [14], as well as the effective connectivity between hippocampus and multiple brain regions related to pain perception and cognitive processing [9], have been found to be altered in migraineurs, with notable differences between EM and CM. Therefore, research focusing on the hippocampus may provide a more comprehensive understanding of the neurobiological mechanisms underlying the chronification of migraine.

Previous neuroimaging studies have shown that patients with migraine typically exhibit reduced hippocampal volume [15, 16]. However, not all studies consistently observe this reduction [17]. A potential explanation for these divergent findings is that existing research has primarily concentrated on total hippocampal volume or voxel-based comparisons within the hippocampus, rather than specifically exploring the volumes of individual subregions. The hippocampus consists of several distinct subregions, each linked to specific neural functions [18]. As such, the aforementioned methodologies may overlook the subtle and differential changes occurring in distinct hippocampal subregions, especially since volume reduction in different subregions may not progress synchronously in pathological states [19]. Many studies combine EM and CM into one migraine group, and given the possibility that EM and CM may exhibit different patterns of hippocampal subregion damage, varying proportions of EM and CM patients across studies can contribute to inconsistent results. In fact, prior research has identified specific volumetric changes in certain hippocampal subregions in patients with migraine with aura [20] and those with medication overuse headache [21]. Additionally, Yu et al. found that the left hippocampal volume in the CM group was smaller than those in the EM group and the healthy control (HC) group, and the volume of hippocampus was negatively correlated with headache frequency [14]. Based on these findings, we initially hypothesized that migraine patients would exhibit volume alterations in specific hippocampal subregions, with some differences between the EM and CM groups.

This research was intended to use the high-resolution hippocampal subregion segmentation to systematically assess the volume differences of hippocampal subregions among the CM, EM and HC groups. Furthermore, we aimed to explore the potential correlations between the volumes of specific hippocampal subregions and migraine-related clinical features.

## Materials and methods

### Subjects and clinical assessment

We prospectively recruited 64 patients with migraine without aura, including 42 EM patients and 22 CM patients, from the Department of Neurology of the First Affiliated Hospital of Soochow University. The diagnosis of migraine was made by an experienced neurologist based on the criteria established in the International Classification of Headache Disorders (ICHD-3 beta) [22]. All migraine patients were without medication overuse. Demographic and clinical data collected for all patients included age, sex, years of education, headache frequency, disease duration, and the 10-point Visual Analogue Scale (VAS) used to assess pain intensity. The patients had no migraine attacks for at least 3 days before and 1 day after MRI scanning. Additionally, 65 HCs balanced for sex, age, and education level were included. None of the HCs had a personal or family history of migraine or mental disorders. All subjects in this study were right-handed. Exclusion criteria for all subjects included migraine with aura; preventive or therapeutic migraine treatments or therapies within 3 days before the MRI scanning; with a history of preventive migraine treatments; any other neurological or psychiatric diseases, including depression, anxiety, impulsivity, and posttraumatic stress disorder; other pain conditions; drug or alcohol abuse; MRI contraindications. The study was carried out according to the Declaration of Helsinki and was approved by the Ethics Committee of the First Affiliated Hospital of Soochow University. All participants provided written informed consents before entering this research.

### MRI acquisition

All subjects were scanned using a 3.0-Tesla MRI system (MAGNETOM Skyra, Siemens Healthcare, Erlangen, Germany) with a 16-channel head and neck joint coil. Foams and earplugs were applied to minimize head movement, and all subjects were asked to remain still, relax, keep eyes closed and stay awake during the scanning. Any subjects with excessive head movement were excluded from the analysis. High resolution T1-weighted anatomic images were acquired for each subject using a sagittal fast spoiled gradient recalled echo sequence: repetition time = 2300 ms, echo time = 2.98 ms, flip angle = 90°, field of view = 256 × 256 mm^2^, matrix = 256 × 256, slice number = 192, slice thickness = 1 mm. After the scanning, two experienced radiologists assessed the structural images to exclude any visible brain lesions.

### Image processing

**Fig. 1:**
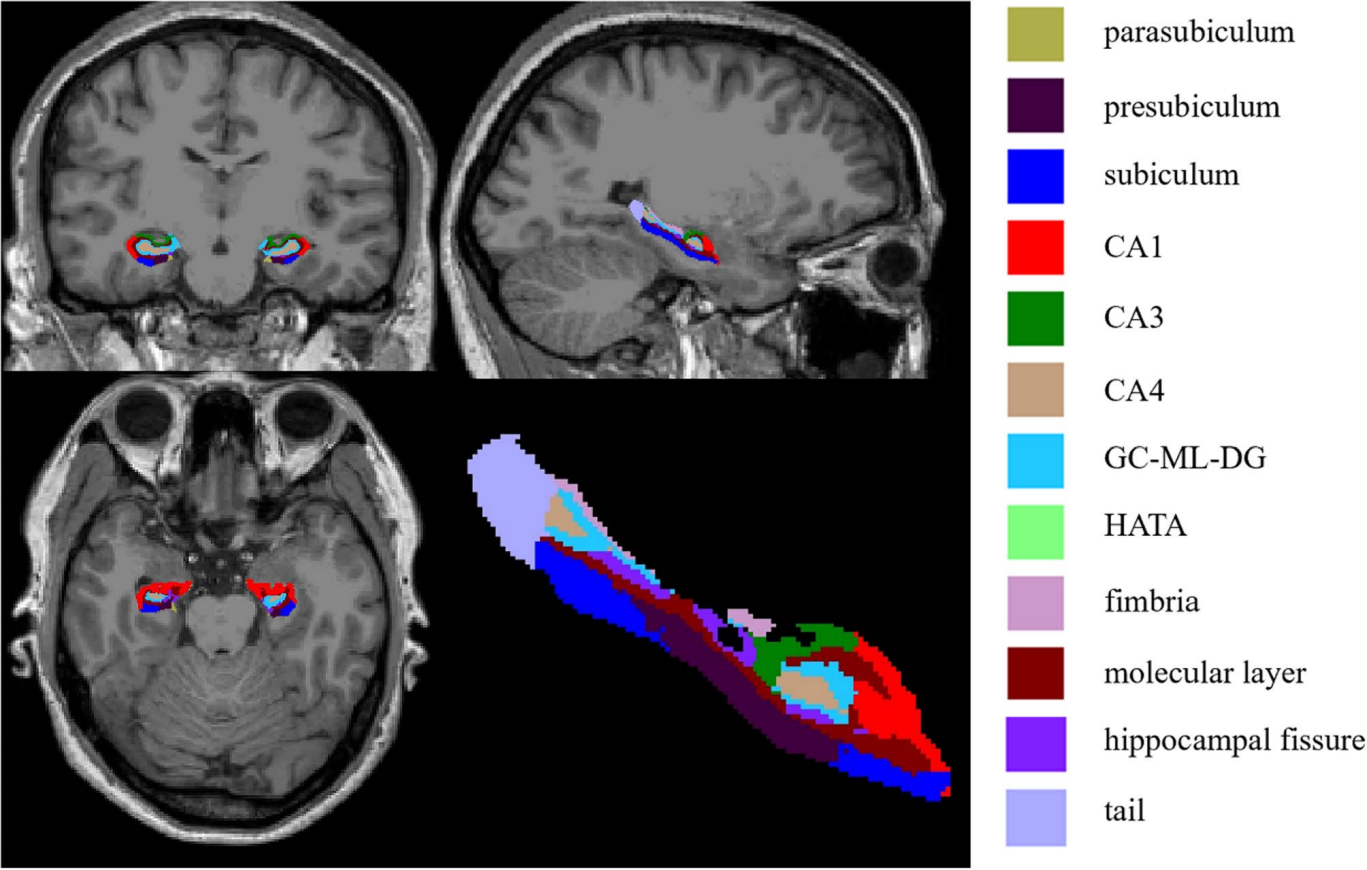
Illustration of hippocampal segmentation. A total of 12 hippocampal subregions were segmented and labeled with different colors. CA, cornu ammonis; GC-ML-DG, granule cell and molecular layer of the dentate gyrus; HATA, hippocampus-amygdala transition area

T1-weighted images were processed through the recon-all script in FreeSurfer v7.3.2 (https://surfer.nmr.mgh.harvard.edu/) for automated cortical reconstruction and segmentation. Details regarding the specific processes have been previously reported [23, 24]. Following the “recon-all” process, the hippocampal segmentation tool was used to divide the hippocampus into its subregions, including hippocampal tail, subiculum, presubiculum, parasubiculum, cornu ammonis (CA) 1, CA3, CA4, hippocampal fissure, molecular layer, molecular layer of the dentate gyrus (GC-ML-DG), hippocampus-amygdala transition area (HATA), and fimbria (Fig. 1) [19]. As previously detailed, the estimated total intracranial volume (eTIV), a measure of the overall intracranial volume used to account for individual differences in head size, is calculated using Freesurfer through the registration of images to the MNI305 Talairach space [25], obtained via the “mri_segstats --etiv-only” command [26]. The volume of each hippocampal subregion and eTIV was obtained for statistical analysis. The hippocampal subregion images were visualized using the FreeView tool package in FreeSurfer.

Regarding the FreeSurfer segmentation outputs, we initially assessed the Euler number to identify and excluded any obvious outliers in the hippocampal subregions [27]. Following this, two junior neuroradiologists conducted preliminary reviews of the segmentations. The final automated segmentation results were then evaluated and approved by a senior neuroradiologist who was blinded to all clinical data. Ultimately, all data included in this study adhered to the established quality control criteria.

### Statistical analysis

The analysis of demographic data, clinical parameters and hippocampal subregion volumes was conducted using the SPSS software (version 27.0, IBM Corp., Armonk, NY, USA). For demographic data, chi-squared test was used to analyze sex distribution, and one-way analysis of variance (ANOVA) was used to assess age and education. For clinical parameters, the Mann–Whitney U test was performed to detect differences between the EM and CM groups. For hippocampal subregion volumes, a general linear model (GLM) analysis was employed to compare differences among the three groups, controlling for age, sex, and eTIV, with false discovery rate (FDR) correction further applied to control for multiple comparisons. Post-hoc analysis of intergroup comparisons was conducted using the least-significant difference method, with FDR correction applied to each hippocampal subregion. Spearman partial correlation analyses, controlling for age, sex, and eTIV, were then used to evaluate associations between hippocampal subregion volumes differing significantly across the three groups and clinical characteristics, including headache frequency, disease duration, and VAS score. Statistical significance was defined as *p* < 0.05, and for multiple comparisons, FDR-corrected *p* (*p*-FDR) < 0.05 were considered significant.

## Results

### Demographic and clinical characteristics

The demographic and clinical indicators of this study are presented in Table 1. There was no significant difference in sex (*p* = 0.746), age (*p* = 0.189), or education (*p* = 0.146) among the three groups. Patients with CM had higher headache frequency (*p* < 0.001), disease duration (*p* = 0.044), and VAS score (*p* = 0.032) than those with EM.

**Table 1:** Demographic and clinical characteristics

	EM (*n* = 42)	CM (*n* = 22)	HC (*n* = 65)	*p* value
Sex (male/female)	15/27	10/12	26/39	0.746^a^
Age (years)	40.88 ± 11.04	44.82 ± 15.74	45.52 ± 13.21	0.189^b^
Education level (years)	10.64 ± 3.84	9.32 ± 4.26	11.51 ± 5.07	0.146^b^
Headache frequency/month	4.18 ± 2.89	20.16 ± 4.52	-	< 0.001^c^
Disease duration (years)	11.02 ± 8.00	16.09 ± 10.28	-	0.044^c^
VAS	6.79 ± 1.46	7.32 ± 1.58	-	0.032^c^

Continuous variables are presented as mean±standard deviation

*EM* episodic migraine, *CM* chronic migraine, *HC* healthy control, *VAS* Visual Analog Scales, *n*, number of subjects

^a^*p* value obtained with Chi-square test

^b^*p* value obtained with one-way analysis of variance

^c^*p* value obtained with Mann–Whitney U test

### Hippocampal subregion volumes

No significant differences in hippocampal subregion volumes were observed among the three groups after FDR correction. However, the GLM revealed trend-level changes in the volumes of several regions, including the left whole hippocampus (*p* = 0.007, *p*-FDR = 0.078), left subiculum (*p* = 0.004, *p*-FDR = 0.078), left CA1 (*p* = 0.038, *p*-FDR = 0.124), left molecular layer (*p* = 0.009, *p*-FDR = 0.078), left GC-ML-DG (*p* = 0.044, *p*-FDR = 0.125), left CA4 (*p* = 0.048, *p*-FDR = 0.125), right whole hippocampus (*p* = 0.013, *p*-FDR = 0.078), right subiculum (*p* = 0.037, *p*-FDR = 0.124), right CA1 (*p* = 0.028, *p*-FDR = 0.121), and right molecular layer (*p* = 0.015, *p*-FDR = 0.078) among the three groups.

**Fig. 2:**
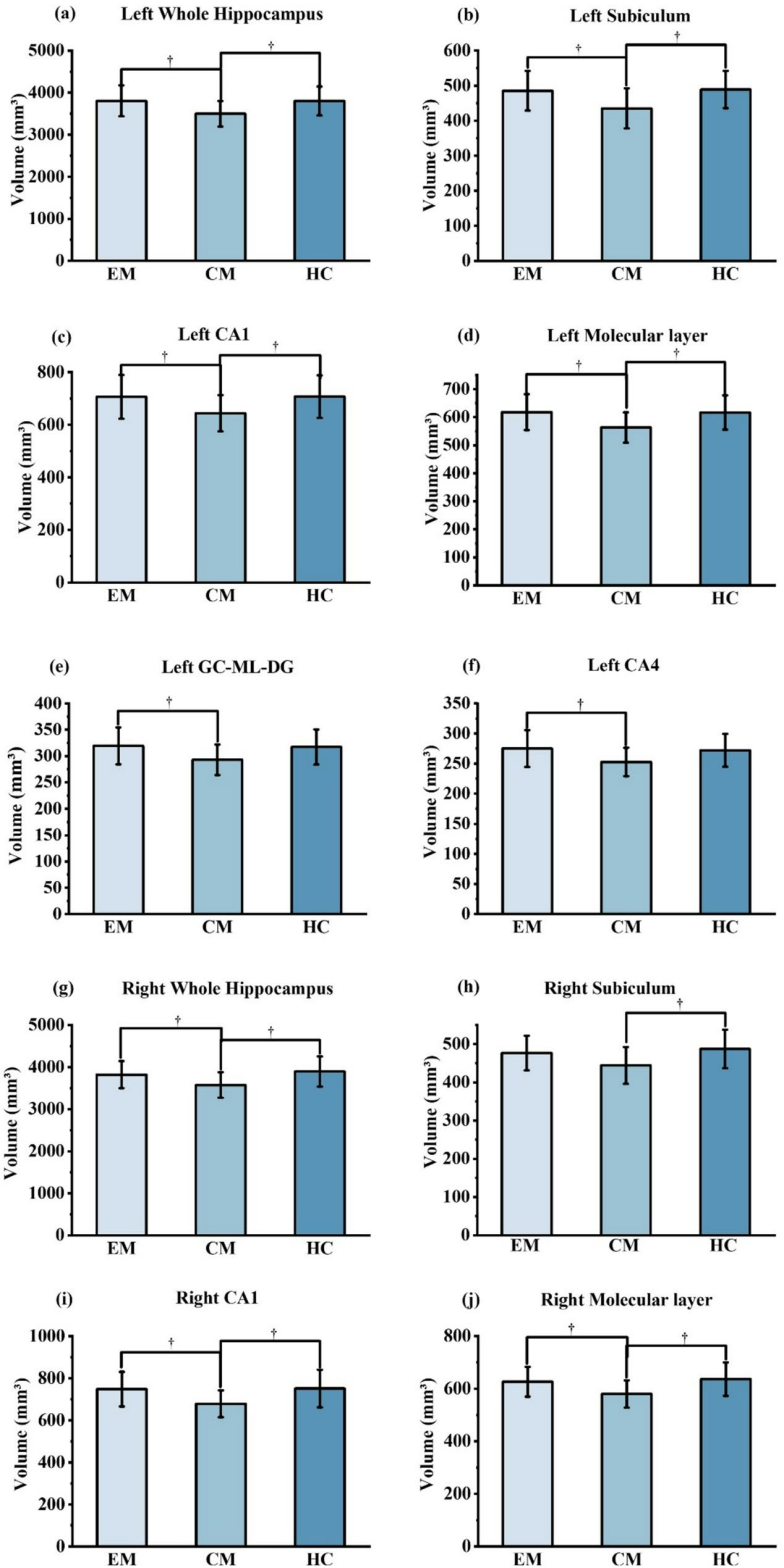
Bar plots show trend-level differences in hippocampal subfield volumes among the EM, CM, and HC groups. Exploratory post-hoc analysis of intergroup comparisons was conducted using the least-significant difference, with FDR correction applied to each hippocampal subregion. Specifically, the comparisons of volumes for the left whole hippocampus **a**, left subiculum **b**, left CA1 **c**, left molecular layer **d**, left GC-ML-DG **e**, left CA4 **f**, right whole hippocampus **g**, right subiculum **h**, right CA1 **i**, and right molecular layer **j** are presented. EM, episodic migraine; CM, chronic migraine; HC, healthy control; CA, cornu ammonis; GC-ML-DG, granule cell and molecular layer of the dentate gyrus; FDR, false discovery rate; *p*-FDR, FDR-corrected *p* † *p*-FDR value less than 0.05

Given that some hippocampal subregions showed significant group differences before FDR correction, exploratory post hoc pairwise comparisons were conducted to examine between-group differences. These post hoc analyses (FDR-corrected) revealed trend-level smaller volumes in the CM group compared to the EM group in several regions, including the left whole hippocampus (*p*-FDR = 0.010), left subiculum (*p*-FDR = 0.010), left CA1 (*p*-FDR = 0.024), left molecular layer (*p*-FDR = 0.010), left GC-ML-DG (*p*-FDR = 0.024), left CA4 (*p*-FDR = 0.024), right whole hippocampus (*p*-FDR = 0.038), right CA1 (*p*-FDR = 0.024), and right molecular layer (*p*-FDR = 0.033). Additionally, the CM group exhibited trend-level smaller volumes in the left whole hippocampus (*p*-FDR = 0.013), left subiculum (*p*-FDR = 0.013), left CA1 (*p*-FDR = 0.030), left molecular layer (*p*-FDR = 0.014), right whole hippocampus (*p*-FDR = 0.013), right subiculum (*p*-FDR = 0.016), right CA1 (*p*-FDR = 0.016), and right molecular layer (*p*-FDR = 0.013) compared to the HC group. No trend-level differences were found between the EM and HC groups in the hippocampal subregions. Table 2 shows detailed results of volume trends of hippocampal subregions among three groups. The trend-level alterations among the three groups after GLM and post-hoc analyses are presented graphically in Fig. 2. The hippocampal volumes for all subregions across the EM, CM and HC groups are shown in Table S1. A graph illustrating the total hippocampal volume changes with age across the EM, CM and HC groups are presented in Fig. S1.

**Table 2:** Trend-level volumetric alterations in hippocampal subregions among the three groups (controlling for age, sex, and estimated total intracranial volume)

	EM (*n* = 42)	CM (*n* = 22)	HC (*n* = 65)	*p* value	*p*-FDR	Exploratory post-hoc analysis
Left Whole Hippocampus	3804.06 ± 366.38	3498.89 ± 304.19	3803.79 ± 342.68	0.007^†^	0.078	EM vs. CM, *p*-FDR = 0.010^†^; EM vs. HC, *p*-FDR = 0.926; CM vs. HC, *p*-FDR = 0.013^†^
Left Subiculum	485.30 ± 56.54	435.00 ± 57.08	489.03 ± 53.23	0.004^†^	0.078	EM vs. CM, *p*-FDR = 0.010^†^; EM vs. HC, *p*-FDR = 0.926; CM vs. HC, *p*-FDR = 0.013^†^
Left CA1	706.02 ± 83.25	643.83 ± 69.09	707.13 ± 80.91	0.038^†^	0.124	EM vs. CM, *p*-FDR = 0.024^†^; EM vs. HC, *p*-FDR = 0.926; CM vs. HC, *p*-FDR = 0.030^†^
Left Molecular layer	617.88 ± 64.09	563.24 ± 53.78	616.64 ± 60.94	0.009^†^	0.078	EM vs. CM, *p*-FDR = 0.010^†^; EM vs. HC, *p*-FDR = 0.926; CM vs. HC, *p*-FDR = 0.014^†^
Left GC-ML-DG	319.36 ± 35.07	292.96 ± 29.06	317.19 ± 33.24	0.044^†^	0.125	EM vs. CM, *p*-FDR = 0.024^†^; EM vs. HC, *p*-FDR = 0.894; CM vs. HC, *p*-FDR = 0.071
Left CA4	274.92 ± 30.59	252.66 ± 23.72	271.99 ± 27.29	0.048^†^	0.125	EM vs. CM, *p*-FDR = 0.024^†^; EM vs. HC, *p*-FDR = 0.894; CM vs. HC, *p*-FDR = 0.098
Right Whole Hippocampus	3822.18 ± 322.50	3574.89 ± 303.41	3896.37 ± 358.71	0.013^†^	0.078	EM vs. CM, *p*-FDR = 0.038^†^; EM vs. HC, *p*-FDR = 0.894; CM vs. HC, *p*-FDR = 0.013^†^
Right Subiculum	476.56 ± 45.30	444.13 ± 48.21	487.12 ± 50.28	0.037^†^	0.124	EM vs. CM, *p*-FDR = 0.054; EM vs. HC, *p*-FDR = 0.894; CM vs. HC, *p*-FDR = 0.016^†^
Right CA1	747.64 ± 82.27	678.24 ± 63.57	750.68 ± 89.43	0.028^†^	0.121	EM vs. CM, *p*-FDR = 0.024^†^; EM vs. HC, *p*-FDR = 0.926; CM vs. HC, *p*-FDR = 0.016^†^
Right Molecular layer	626.35 ± 56.57	580.15 ± 51.81	636.08 ± 63.42	0.015^†^	0.078	EM vs. CM, *p*-FDR = 0.033^†^; EM vs. HC, *p*-FDR = 0.894; CM vs. HC, *p*-FDR = 0.013^†^

Hippocampal subregion volume data are presented as mean±standard deviation values in cubic millimeters. Exploratory post-hoc analysis of intergroup comparisons was conducted using the least-significant difference, with FDR correction applied to each hippocampal subregion

*EM* episodic migraine, *CM* chronic migraine, *HC* healthy control, *GC-ML-DG* granule cell layer of the dentate gyrus, *CA* cornu ammonis, *n* number of subjects, *FDR* false discovery rate, *p-FDR* FDR-corrected *p*

^†^*p* value or *p*-FDR value less than 0.05

### Correlation analysis

Since no significant volumetric differences in hippocampal subregions were observed among the three groups, correlation analysis was not conducted in our study.

## Discussion

In this preliminary study, we applied FreeSurfer-based hippocampal subregion segmentation to examine volumetric differences among CM, EM and HC groups. No statistically significant differences in hippocampal subregion volumes were observed across the three groups. Nevertheless, exploratory analyses revealed trend-level alterations: (1) compared with HCs, patients with CM exhibited trend-level smaller volumes in the bilateral whole hippocampus, subiculum, CA1, and molecular layer; and (2) compared with the EM group, CM patients showed trend-level reductions in the bilateral whole hippocampus, molecular layer, CA1, as well as in the left subiculum, GC-ML-DG, and CA4. Overall, these analyses provide preliminary indications of trend-level volumetric alterations in hippocampal subregions among CM patients.

Similar to our findings, a previous study also reported no statistically significant differences in the left and right hippocampal volumes among CM, EM, and HC groups [28]. The lack of statistically significant differences in our study may be explained by several factors, including limited sample size, individual variability, and the resolution and sensitivity constraints of MRI. In addition, it has been demonstrated that a hippocampal subregion volume exceeding 300 mm³ offers the best test-retest reliability and stability [29], implying that smaller hippocampal volumes in our study might have lower measurement reliability. Indeed, some hippocampal subregions have been excluded or merged in previous studies [30–32]. Furthermore, the anatomical boundaries of small subregions such as CA3, GC-ML-DG, and the molecular layer are not well characterized [33], which may further compromise accuracy. Taken together, the absence of significant findings in our study may be explained by the combined influence of multiple factors. In this study, we reported the original results while acknowledging the need for future research with larger samples, optimized imaging protocols, and improved subregion segmentation strategies.

Despite the absence of statistically significant findings, trend-level variations in hippocampal subregion volumes were observed across the three groups. In general, patients with CM tended to show reduced hippocampal volumes compared with the other two groups, a pattern that is partly consistent with previous reports [10, 14]. As a structure involved in the perception and modulation of pain, the hippocampus is linked to susceptibility to chronic pain [34]. It has been demonstrated that patients with CM have abnormalities in both the structure and functional connectivity of the hippocampus [35, 36]. One possible explanation is that chronic pain-induced stress influences the hypothalamic-pituitary-adrenal axis, leading to elevated glucocorticoids, which may inhibit hippocampal neurogenesis and influence hippocampal remodeling, ultimately causing hippocampal volume reduction [37–39]. Additionally, neuroinflammatory mechanisms play a crucial role in the pathogenesis of CM, which can affect hippocampal neurogenesis and synaptic plasticity, and lead to memory impairment [40, 41]. Considering that memory dysfunction could be a symptom in CM patients [42], it is plausible to suggest that the observed trend-level reductions in hippocampal volumes might serve as a mediator between neuroinflammation and memory dysfunction in CM.

The hippocampus is a highly complex and heterogeneous brain structure, with each subregion serving distinct functional roles. Specifically, the CA1 plays a crucial role in emotion regulation and memory, and its volume reduction has been found to be correlated with memory scores [43]. Furthermore, this subregion forms a functional circuit with the medial prefrontal cortex, which is essential for encoding and retrieving episodic memories [44]. Besides the aforementioned memory issues in CM, co-occurring emotional problems are also common in CM patients and may exacerbate headaches [45]. Therefore, we tentatively suggest that the trend-level involvement of CA1 may be linked to both memory and emotional dysfunctions associated with CM. The CA1/subiculum is a major anatomical connection area for several cortical regions, including the hypothalamus, which is regarded as one of the primary migraine generators [20]. Altered connectivity between the hypothalamus and other brain regions, along with its correlation to the severity of headache, has been observed in patients with CM [46]. Consequently, the trend-level alterations observed in the subiculum, as the primary output region of the hippocampus, may suggest its potential involvement in the physiological mechanisms underlying migraine chronification through its connectivity with the hypothalamus. The molecular layer, composed of interneuron synaptic connections, plays a crucial role in hippocampal synaptic circuitry and the temporal processing of events [47]. Considering that chronic pain is closely associated with abnormal changes in synaptic connections [48], we assume that the observed decreased trend-level molecular layer volumes in CM patients may be associated with impaired synaptic transmission and related pain processing issues.

Moreover, it was found that CM patients exhibited a trend-level reduction in the volume of CA4/DG region compared to EM patients. The CA4/DG can induce neurogenesis and drive neuronal plasticity, with neurogenesis being considered one of the key factors in maintaining cognitive function [49]. Noorani et al. showed that the volume of the CA4/DG in patients with trigeminal neuralgia, a chronic pain disorder, was reduced and subsequently recovered after pain relief achieved through surgical interventions [50], suggesting the CA4/DG region serves as a biomarker for changes in neuronal plasticity and neurogenesis associated with pain. Accumulating evidence [42, 51, 52] indicates that cognitive deficits are common in CM, potentially linked to intracranial pathological changes resulting from recurrent migraine attacks [53]. Based on the above observations, we tentatively suggest that the trend-level reduced volume of CA4/DG in CM patients may be related to their long-standing chronic pain, which might hinder neurogenesis and could be linked to cognitive dysfunction.

It is worth noting that the trend-level changes in hippocampal subregions in CM patients were primarily concentrated in the left hemisphere. Similar reports of left hippocampus involvement have been described in prior CM studies using voxel-based morphometry [10] and resting-state functional connectivity [54]. Taken together, these observations suggest that the hippocampal abnormalities associated with migraine chronification may demonstrate a left-sided lateralization. Indeed, it has been recognized that the left hippocampus plays a predominant role in verbal memory, whereas the right hippocampus is more closely associated with visuospatial memory [55]. According to a clinical investigation, CM may primarily affect verbal memory [56], which is predominantly associated with the left hippocampus. Therefore, it is not surprising that trend-level alterations in CM were observed mainly in the left hippocampus. However, it should also be noted that the trend-level reduction in the CA1, molecular layer, and subiculum subregions in CM patients occurred in both hemispheres. This bilateral reduction cannot be conclusively explained by the current study design. One tentative interpretation is that, while CM has been reported to primarily affect verbal memory, it may also impact other functions related to the right hippocampus. The left-sided lateralization, together with the partial bilateral involvement observed, may be related to the multifaceted and widespread functional impact of migraine chronification. Nonetheless, as our study did not collect the relevant scales about memory as well as other cognitive functions, these interpretations remain speculative and require further validation in future research.

Interestingly, the present study showed no trend-level alterations in hippocampal subregion volumes between the EM and HC groups. As seen in Fig. 2, in the subregions exhibiting trend-level differences across the three groups, the EM and HC groups had comparable volumes, whereas the CM group appeared to show a trend-level reduction. One explanation could be that, during the EM phase of migraine (with a relatively low frequency of headache attacks), hippocampal subregions may undergo compensatory adaptive changes through neuroplastic mechanisms, which may help preserve subregion volumes at this stage. As the condition progresses to the CM phase, however, these adaptive changes may become maladaptive, contributing to the trend-level reductions in hippocampal subregion volumes observed in CM. Therefore, this observation may underscore the potential importance of early intervention in EM to reduce the risk of progression to CM in terms of hippocampal subregion alterations.

Our research had several limitations. First, one limitation of this study is the relatively small and unequal sample size, particularly for CM group, which is due to its lower prevalence compared to EM in the general population. The overall smaller sample size increases the risk of Type II errors, meaning that we may miss some hippocampal subregions that are potentially significant. This limitation should be considered when interpreting the findings, and future research with larger and more balanced samples is needed to confirm these results. Second, the participant groups were not perfectly sex- or age- matched, but rather balanced. Future research should focus on achieving a more optimal sex ratio and a closer age match between groups to enhance the robustness of the results. Third, as the participants were recruited from a single center, the generalizability of the results requires further validation using a multicenter dataset. Fourth, as previously mentioned, one study suggests that hippocampal subregion volumes exceeding 300 mm³ provide the best test-retest reliability and stability [29], meaning that caution should be taken when evaluating our measurements of smaller hippocampal subregions. Fifth, some patients had a history of using acute-phase therapeutic medications (e.g., non-steroidal anti-inflammatory drugs), but the specific types and dosages of these medications were not thoroughly recorded. This represents an important limitation that should be further explored and addressed in future research. Last, it is important to note that our study is only a cross-sectional study, and therefore it is not yet possible to infer any causal relationships. In the future, prospective longitudinal studies tracking the dynamic changes are needed to address this issue.

In conclusion, this study did not identify statistically significant volumetric differences in hippocampal subregions among the three groups. However, exploratory analyses revealed trend-level reductions in several subregions in CM patients, including CA1, CA4, molecular layer, GC-ML-DG, and subiculum. These preliminary observations may suggest potential—but unconfirmed—associations between hippocampal subregional characteristics and processes such as pain perception, emotion, memory, and cognition during migraine chronification. Future studies with larger, well-balanced samples and higher-resolution imaging are required to clarify these tentative findings.

## Supplementary Information

Supplementary material 1.

## Data Availability

The datasets used and/or analysed during the current study are available from the corresponding author on reasonable request.

## Abbreviations

ANOVA: one-way analysis of variance
CA: cornu ammonis
CM: chronic migraine
EM: episodic migraine
eTIV: estimate total intracranial volume
FDR: false discovery rate
GC-ML-DG: granule cell and molecular layer of the dentate gyrus
GLM: general linear model
HATA: hippocampus-amygdala transition area
HC: healthy control
*p*-FDR: FDR-corrected *p*
VAS: Visual analog scales
