# Supplementary material for: Volumetric analysis of hippocampal subregions in migraine without aura: an exploratory study on mechanisms underlying migraine chronification
Source: J Headache Pain. 2025 Oct 31;26(1):236. doi: 10.1186/s10194-025-02183-0 (PMC12577149; doi:10.1186/s10194-025-02183-0)
Supplement: Supplementary file 1 — Supplementary material 1. [file 10194_2025_2183_MOESM1_ESM.docx]

**Supplementary Material**

**Table S1.** Hippocampal subregion volumes across the three groups (controlling for age, sex, and estimated total intracranial volume)

|  | EM (*n*=42) | CM (*n*=22) | HC (*n*=65) | p value |
| --- | --- | --- | --- | --- |
| Left Whole Hippocampus | 3804.06±366.38 | 3498.89±304.19 | 3803.79±342.68 | 0.007^†^ |
| Left Hippocampal tail | 625.27±76.07 | 625.27±76.07 | 625.27±76.07 | 0.135 |
| Left Subiculum | 485.30±56.54 | 435.00±57.08 | 489.03±53.23 | 0.004^†^ |
| Left CA1 | 706.02±83.25 | 643.83±69.09 | 707.13±80.91 | 0.038^†^ |
| Left Hippocampal fissure | 156.33±30.61 | 140.61±24.74 | 162.86±30.09 | 0.069 |
| Left Presubiculum | 333.00±48.83 | 307.91±44.48 | 332.89±44.02 | 0.237 |
| Left Parasubiculum | 63.61±13.06 | 65.72±171.5 | 64.67±14.31 | 0.563 |
| Left Molecular layer | 617.88±64.09 | 563.24±53.78 | 616.64±60.94 | 0.009^†^ |
| Left GC-ML-DG | 319.36±35.07 | 292.96±29.06 | 317.19±33.24 | 0.044^†^ |
| Left CA3 | 231.29±31.32 | 216.09±26.39 | 230.43±30.41 | 0.411 |
| Left CA4 | 274.92±30.59 | 252.66±23.72 | 271.99±27.29 | 0.048^†^ |
| Left Fimbria | 87.31±23.29 | 80.67±22.29 | 83.32±17.04 | 0.407 |
| Left HATA | 60.11±9.98 | 56.38±7.04 | 61.56±11.16 | 0.342 |
| Right Whole Hippocampus | 3822.18±322.50 | 3574.89±303.41 | 3896.37±358.71 | 0.013^†^ |
| Right Hippocampal tail | 639.11±79.48 | 617.01±79.87 | 660.69±70.92 | 0.111 |
| Right Subiculum | 476.56±45.30 | 444.13±48.21 | 487.12±50.28 | 0.037^†^ |
| Right CA1 | 747.64±82.27 | 678.24±63.57 | 750.68±89.43 | 0.028^†^ |
| Right Hippocampal fissure | 159.51±30.95 | 150.90±29.35 | 166.01±30.01 | 0.641 |
| Right Presubiculum | 311.08±36.68 | 288.85±38.57 | 316.51±35.43 | 0.113 |
| Right Parasubiculum | 57.77±11.10 | 57.16±11.85 | 60.45±10.68 | 0.603 |
| Right Molecular layer | 626.35±56.57 | 580.15±51.81 | 636.08±63.42 | 0.015^†^ |
| Right  GC-ML-DG | 315.89±29.57 | 297.21±24.83 | 322.38±33.39 | 0.102 |
| Right CA3 | 237.57±30.10 | 230.01±25.68 | 247.69±33.59 | 0.351 |
| Right CA4 | 268.14±23.59 | 255.87±21.08 | 276.56±29.51 | 0.141 |
| Right Fimbria | 80.55±17.78 | 69.08±19.42 | 75.28±17.82 | 0.104 |
| Right HATA | 61.52±9.69 | 58.33±8.66 | 61.29±8.68 | 0.834 |

**Note:** Hippocampal subregion volume data are presented as mean±standard deviation values in cubic millimeters.

EM, episodic migraine; CM, chronic migraine; HC, healthy control; GC-ML-DG, granule cell layer of the dentate gyrus; CA, cornu ammonis; HATA, hippocampus-amygdala transition area; *n*, number of subjects

^†^ p value less than 0.05


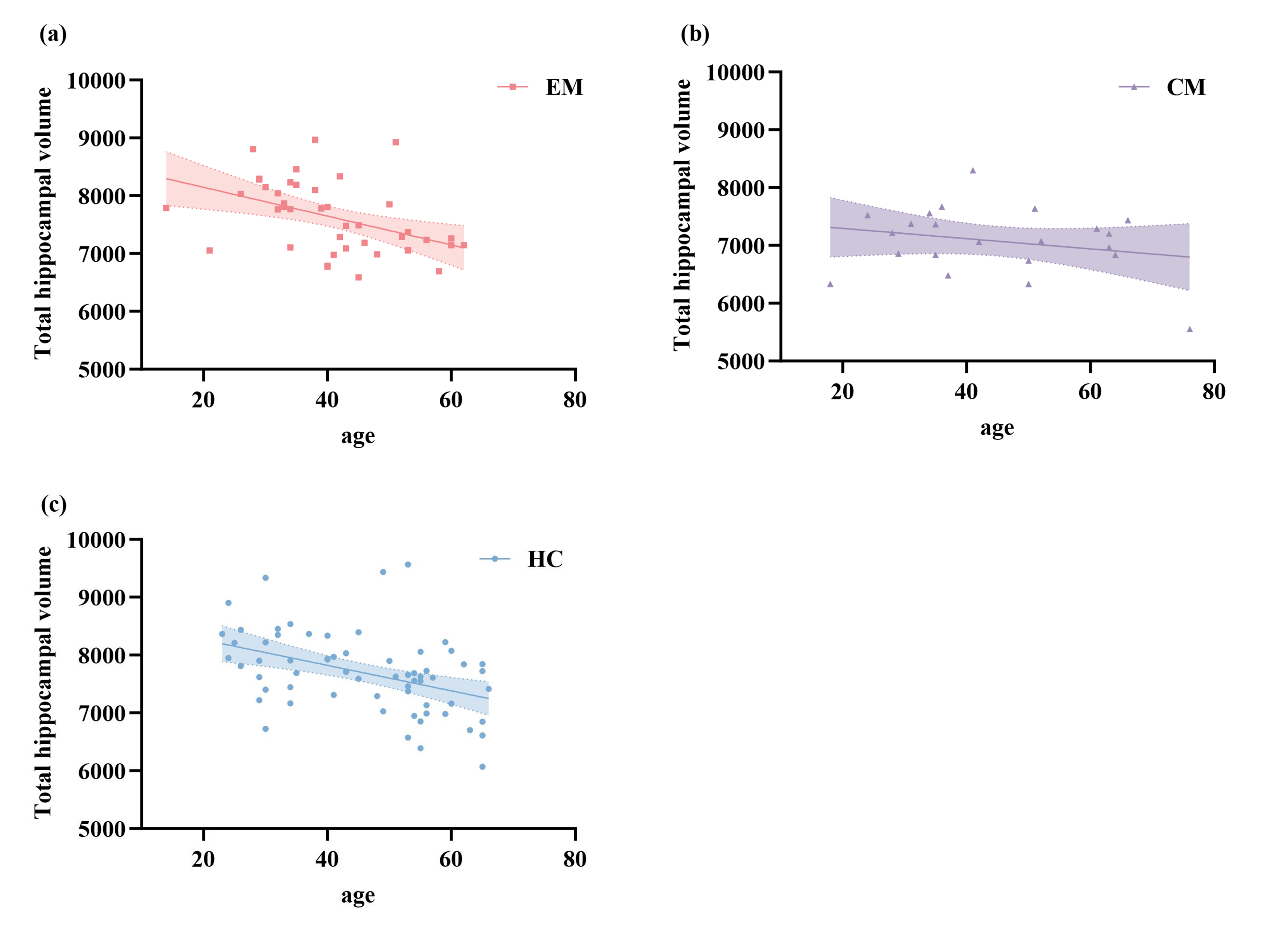


**Fig. S1.** Graph of hippocampal volume changes with age across the EM (a), CM (b) and HC (c) groups. As shown in the figure, total hippocampal volume declined with age across all three groups, with the CM group exhibiting a more gradual decline.

EM, episodic migraine; CM, chronic migraine; HC, healthy control
